# The possible pathogenesis of macular caldera in patients with North Carolina macular dystrophy

**DOI:** 10.1186/s12886-022-02655-w

**Published:** 2022-11-19

**Authors:** Zhe Zhu, He Zou, Chuanyu Li, Bainan Tong, Chenchen Zhang, Jun Xiao

**Affiliations:** 1 Medical Retina, Eye Center of the Second Hospital of Jilin University, Room 304, 3Rd Floor, Out Patient Building, No.218, Ziqiang Street, Nanguan District, Changchun City, Jilin Province China; 2 Eye Center of the Second Hospital of Jilin University, Changchun City, Jilin Province China

**Keywords:** North Carolina macular dystrophy, Chinese family, PRDM13, Pathogenesis

## Abstract

**Background:**

This study provides a detailed description of a Chinese family with North Carolina macular dystrophy (NCMD) and explores its possible pathogenesis.

**Methods:**

Five individuals from a three-generation family underwent general ophthalmic examination, multi-imaging examinations and visual electrophysiology examinations when possible. Genetic characterization was carried out by target region sequencing and high-throughput sequencing in affected patients.

**Results:**

Despite severe fundus changes, patients had relatively good visual acuity. Genetic analysis showed that affected patients had PRDM13 gene duplication and heterozygous mutations of the ABCA4 gene. Optical coherence tomography (OCT) showed an abnormal retinal pigment epithelium (RPE) layer in patients with grade 2 lesions, while the neurosensory retina was relatively normal. In grade 3 patients, RPE and choroid atrophy were greater than that of the neurosensory retina, showing concentric atrophy.

**Conclusions:**

RPE and choroidal atrophy were found to play an important role in the development of macular caldera.

## Background

North Carolina macular dystrophy (NCMD), first reported in 1971 in North Carolina by Lefler et al., is an autosomal dominant retinal macular dystrophy. Three subtypes of this disease have been described: MCDR1, MCDR2 and MCDR3 [1, 2]. MCDR1 has been the most intensively analysed, with the disorder being mapped to chromosome 16q16 in multiple families [3–5]. Current research suggests that PRDM13 genes are likely to be responsible for MCDR1 [6]. NCMD can be divided into three grades according to the different manifestations of fundus lesions. Grade 1–2 fundus lesions tend to manifest as drusen-like lesions, while grade 3 lesions exhibit macular caldera [7]. The specific pathogenesis of NCMD is, nevertheless, still unclear.

There have been few reports of NCMD in an Asian population [8, 9]. The purpose of this research is to provide a detailed description of a Chinese family with NCMD to help better understand NCMD and to explore its possible pathogenesis.

## Methods

**Fig. 1:**
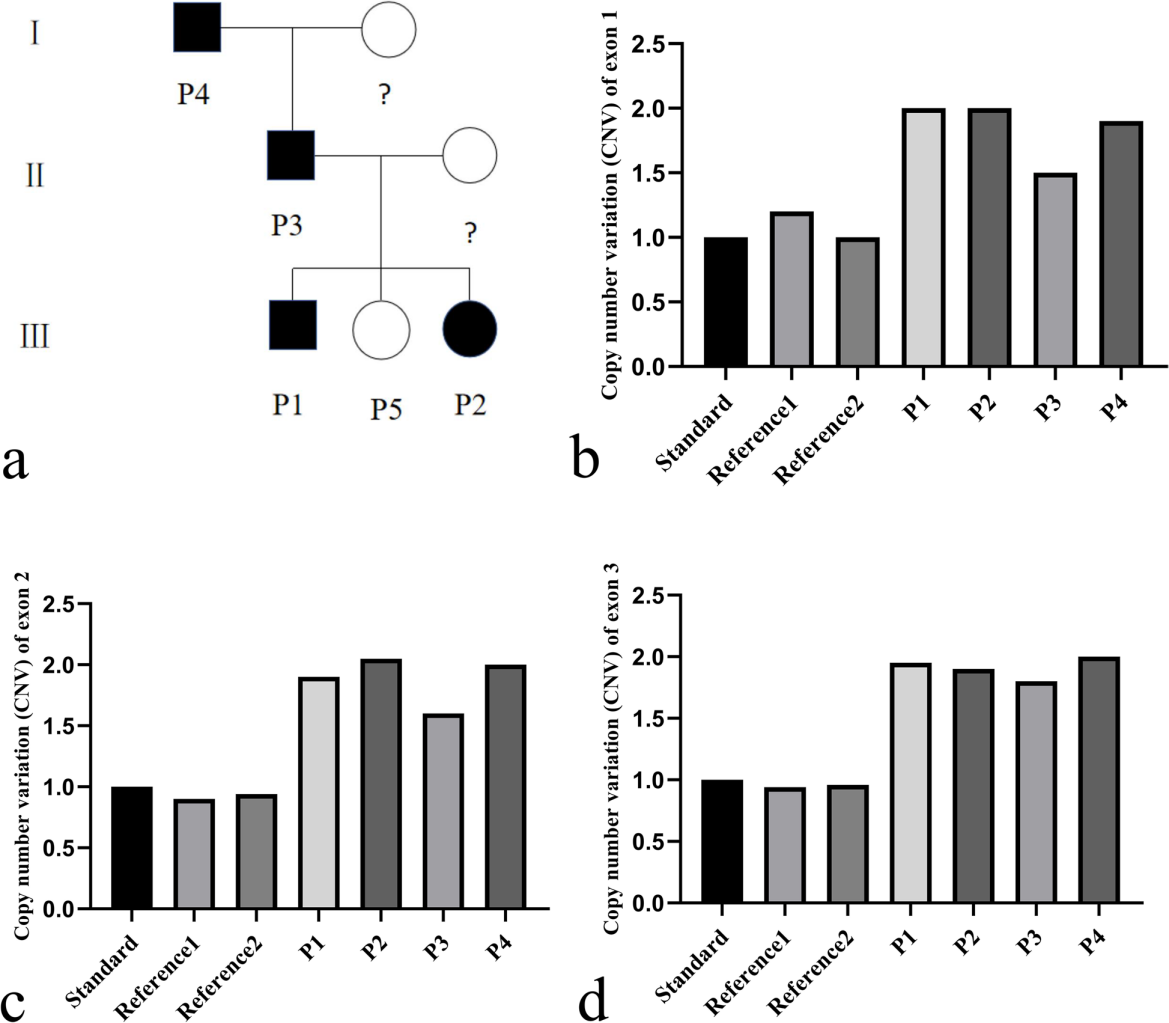
**a** Pedigree chart of a Chinese family affected by North Carolina macular dystrophy (NCMD). Question marks indicate that the family member has not been examined; (**b**-**d**) show the duplication of exons 1, 2, and 3 in the PRDM13 gene of P1-4, respectively, which were confirmed by quantitative real-time polymerase chain reaction

We retrospectively reviewed five individuals (P1, P2, P3, P4 and P5; shown in Fig. 1a) in three generations of a Chinese family who were examined and diagnosed at Eye Center of the Second Hospital of Jilin University. The patients (10 eyes; 5 patients) included in this study were assessed by means of a general ophthalmic examination, and multi-imaging procedures such as fundus photography, spectral-domain optical coherence tomography (SD-OCT, Heidelberg Engineering), and fundus autofluorescence (FAF) were performed when possible. Multifocal electroretinogram (mfERG), full-field electroretinogram (ERG), and visual evoked potential (VEP) data were also obtained from P2-4.

EDTA whole blood samples from 4 affected family members (P1-4) were collected for genetic analysis, performed by target region sequencing and high-throughput sequencing.

## Results

### General clinical manifestations

P1 is the proband, a boy who was noted to have a mottled macular pigment 10 days after birth, during new-born eye screening. P2 is a 16-year-old female, P1’s elder sister, who experienced poor vision since childhood. She had attended our hospital for an ophthalmological evaluation 6 years earlier but was not diagnosed with NCMD at that time. The visual acuity (VA) of P2 was 20/100 OD and 20/125 OS, and the intraocular pressure (IOP) was 18 mmHg OD and 16 mmHg OS. Exotropia was 10 degrees in the left eye, and the anterior segments of both eyes were normal. P3 is a 40-year-old man, the father of the proband; he had experienced poor vision since childhood but was not diagnosed or treated. He experienced no obvious change in vision. The VA of P3 was 20/63 OD and 20/50 OS, IOP was 16 mmHg OD and 19 mmHg OS, and both anterior segments were normal. P4 is a 68-year-old man, the grandfather of P1, who had experienced poor vision since childhood without diagnosis or treatment. The VA of P4 was 20/63 OD and 20/50 OS, IOP was 15 mmHg OD and 11 mmHg OS, and both anterior segments were normal. P5 is an 8-year-old girl, another elder sister of P1. The VA of P5 was 20/20 OD and 20/50 OS, and both anterior segments and ocular fundus were normal.

### Genetic analysis

**Fig. 2:**
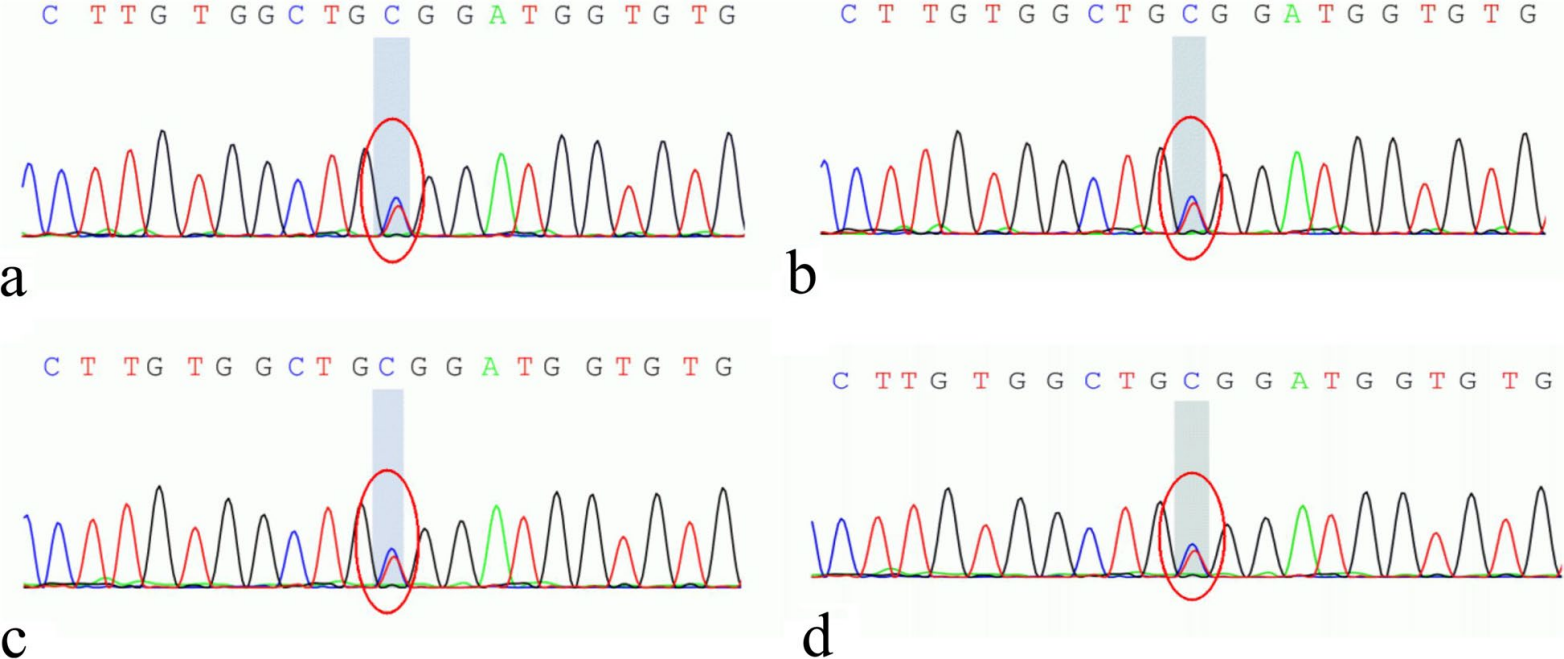
**a**-**d** show the heterozygous mutations in the ABCA4 gene (mutation information: c.4103G > A, chr1-94,497,359, p. R1368H) of P1-4, respectively, which were confirmed by Sanger sequencing

According to previous studies, MCDR1 can be caused by dysregulation of the retinal transcription factor PRDM13 [6]. High-throughput sequencing showed that the PRDM13 gene (containing 4 exons) may have manifested full gene duplication in P1-4, among which the duplication of exons 1–3 was confirmed by quantitative real-time polymerase chain reaction (qPCR, Fig. 1b-d). In addition, Sanger sequencing identified heterozygous mutations in the ABCA4 gene in P1-4 (mutation information: c.4103G > A, chr1-94,497,359, p.R1368H, Fig. 2a-d). This mutation is seen in many retinal degenerative diseases, such as Stargardt disease-1 (STGD1), and shows STGD1-like features. MCDR2 combined with a heterozygous ABCA4 mutation has been previously reported by Lee W et al., but the patients reported here did not exhibit distinct STGD1-like features (Fig. 4a-c) [10]. This may be because STGD1 is an autosomal recessive disease, and the patients in this study had heterozygous mutations.

### Multi-imaging

**Fig. 3:**
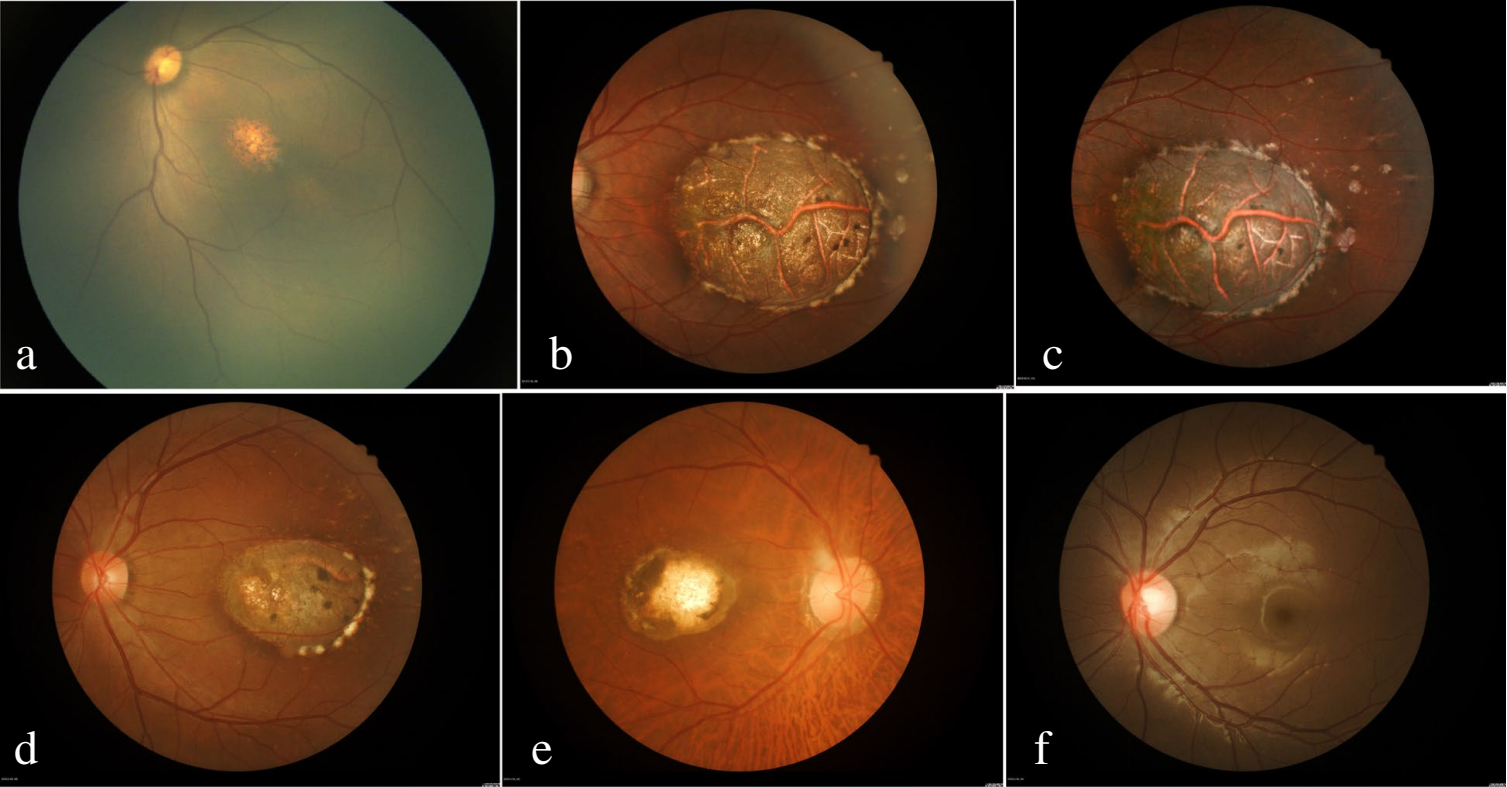
**a**, **b**, **d**, **e** sequentially show the fundus photographs of P1-4; (**c**) c is the fundus photograph of P2 taken in October 2012, 6 years before **b**; (**f**) the left eye of P5 was normal

**Fig. 4:**
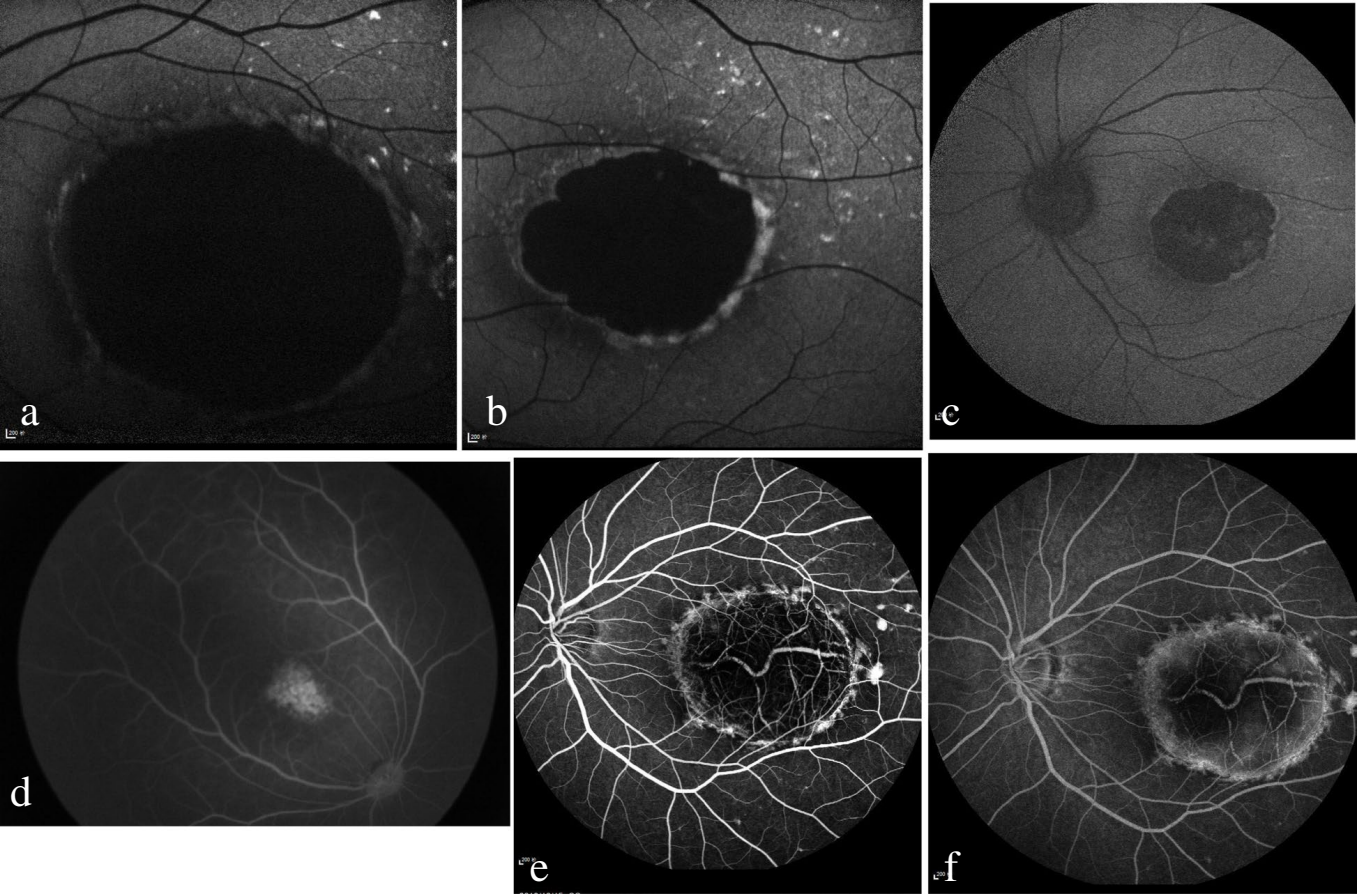
**a**-**c** shows the fundus autofluorescence of P2-4 in turn; **d** shows the early fundus fluorescein angiography (FFA) stage of P1; (**e**, **f**) are the early and late FFA stages of P2, respectively

Part of the fundus image data for P2 was obtained in October 2012. All other image data were collected in January 2019. Fundus photographs revealed orange‒yellow, confluent, drusen-like lesions in the fovea region of P1’s eyes, while the eyes of P2-4 presented with macular caldera and visible sclera. Thus, P1 had grade 2 lesions, and P2-4 had grade 3 lesions (Fig. 3a, b, d, e) [7]. At the same time, typical greyish-white, accumulated subretinal tissue was found at the edge of the macular atrophy in P2-4. Pigmentation could be identified in the lesions, and drusen-like, yellow‒white dot deposits were seen around the macular atrophy (Fig. 3b, d, e). A comparison of the fundus imaging data of P2 obtained in 2012 with those obtained in 2019 revealed that the fundus lesions of P2 had not changed in 6 years (Fig. 3b, c). Furthermore, the fundus photographs of both eyes of P5 showed no abnormal manifestations (Fig. 3f). In addition, the FAF of P2-4 showed the absence of fluorescence in macular lesions, indicating the absence of retinal pigment epithelium (RPE). The edge of the lesions and the yellow‒white dot deposits exhibited high fluorescence (Fig. 4a-c). A fundus fluorescein angiography (FFA) examination of P1 showed sheet transparent fluorescence in the macular region of both eyes. On the other hand, P2 exhibited oval retinal defects in both eyes at the early stage, with fluorescence of the choroidal vessels, high fluorescence at the edge of the lesions, dot-like high fluorescence around the lesions, and fluorescence staining in the macular region at the late stage (Fig. 4d-f).

**Fig. 5:**
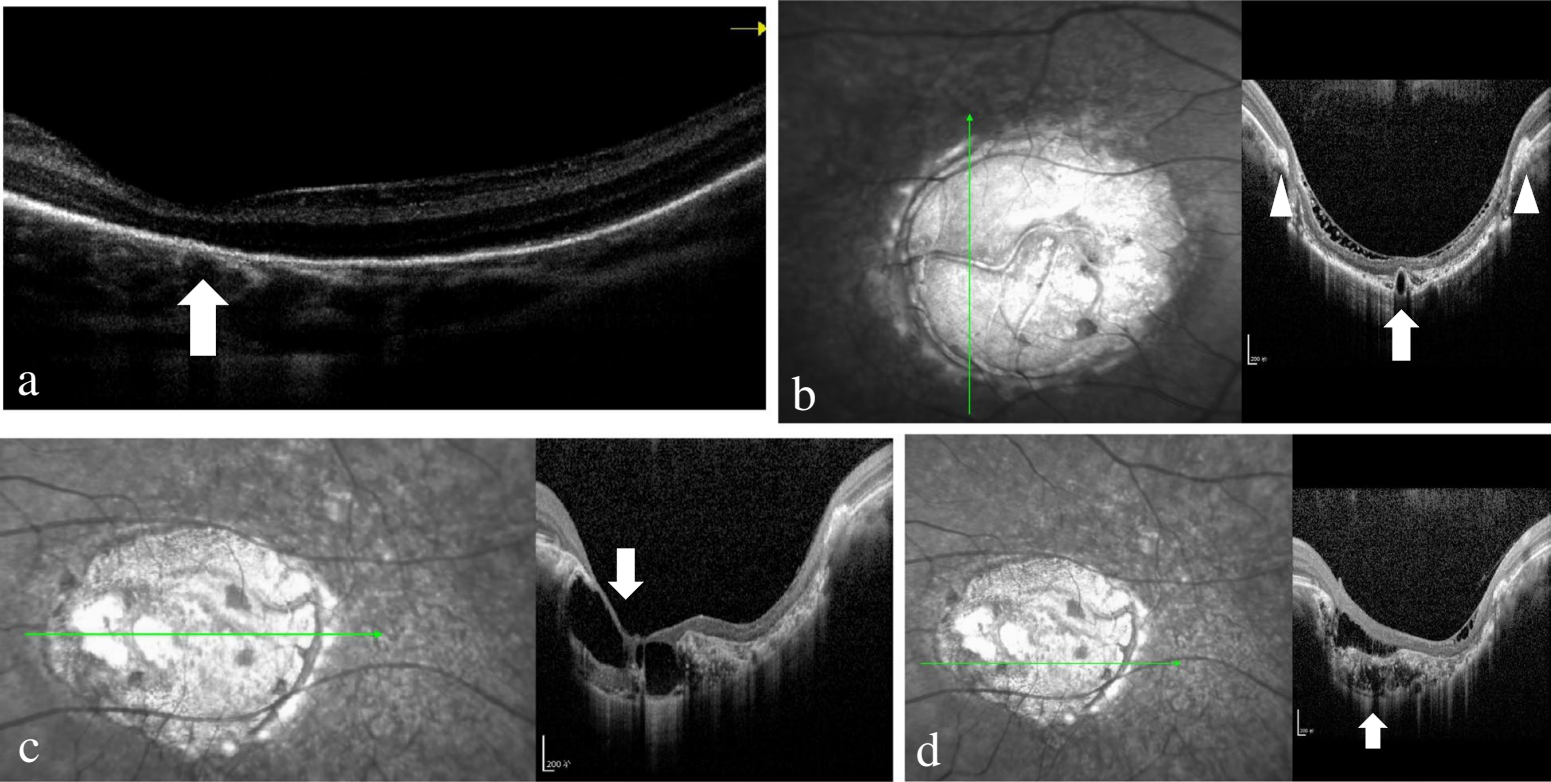
**a** Optical coherence tomography (OCT) of P1’s left eye showed an abnormal retinal pigment epithelium (RPE) layer in the macula (arrow); (**b**) OCT of P2’s left eye revealed choroid macrovessels (arrow) and subretinal hyperreflective tissues at the edge of the lesion (arrowhead); (**c**) OCT of P3’s left eye showed nonreflective cavities (arrow); (**d**) OCT of P3’s left eye showed hyperreflective substances under the neurosensory retina (arrow)

**Fig. 6:**
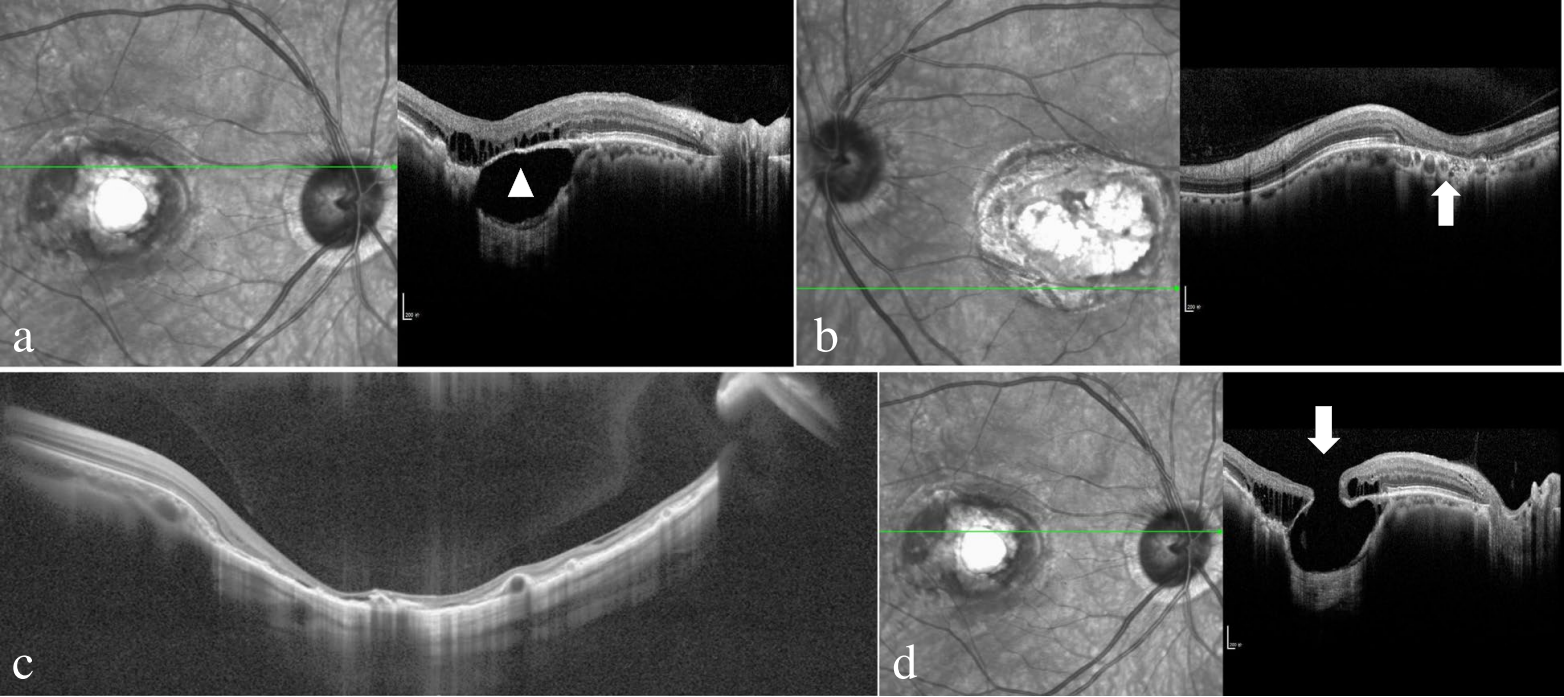
**a** P4’s right eye. Choroid atrophy was observed at the edge of the lesion, and the neurosensory retina and RPE were preserved (arrowhead). **b** P4’s left eye. RPE atrophy was observed at the edge of the lesion, and the neurosensory retina and choroid were preserved (arrow). **c** P2’s left eye. The remaining covering of the neurosensory retinal layer in the lesion can be observed. **d** P4’s right eye. Note the macular hole changes (arrow)

Optical coherence tomography (OCT) of P1 revealed an abnormal RPE layer in the macular region of both eyes, although the neurosensory retina was relatively normal (Fig. 5a). P2-4 had macular caldera lesions, while OCT revealed complete atrophy of the choroidal structure. Only a few large choroid blood vessels attached to the sclera and/or hyperreflective substances beneath the neurosensory retina were retained. The broken ends of the RPE and subretinal hyperreflective tissues represented in the fundus photography could be seen at the edge of the lesion. We also observed nonreflective cavities underneath the neurosensory retina (Fig. 5b-d) [11]. By careful observation of the OCT in P2-4, we were able to detect RPE and choroid atrophy at the edge of grade 3 lesions, while the neurosensory retinal layer remained relatively intact. The RPE and choroid in the lesions were completely atrophied, and the neurosensory retina was also atrophied and thinned, but some areas remained covered. Atrophy appeared in a pattern of concentric circles, so the area of RPE and choroid atrophy was larger than that of the neurosensory retina. This feature has also been shown in OCT in previous studies but has not been described in detail (Fig. 6a-c) [7, 12]. In addition, OCT in the right eye of P4 was associated with macular hole changes (Fig. 6d), and OCT in P5 showed no abnormal changes.

### Electrophysiology of vision

Full-field ERG in P2-4 appeared normal but showed reduced mfERG amplitude within the lesion area and delayed mfERG implicit time in the macular area. The VEP data in P2-4 were normal (Table 1).

**Table 1:** Case presentations

Patient	Age	Sex	Visual acuity		Clinical findings (Grade)	Nonreflective cavities	Macular hole	Whole gene duplication in PRDM13 gene	Heterozygous mutations in ABCA4 gene	Full field ERG	mfERGs
			Right eye	Left eye							
P1	10 dys	Male	N	N	confluent drusen-like lesions (grade 2)	N	N	Y	Y	Patient refused	Patient refused
P2	16 yrs	Female	20/100	20/125	macular caldera (grade 3)	Y	N	Y	Y	Normal	Abnormal
P3	40 yrs	Male	20/63	20/50	macular caldera (grade 3)	Y	N	Y	Y	Normal	Abnormal
P4	68 yrs	Male	20/63	20/50	macular caldera (grade 3)	Y	Y	Y	Y	Normal	Abnormal
P5	8 yrs	Female	20/20	20/50	Normal	N	N	Patient refused	Patient refused	Patient refused	Patient refused

*N* No, *Y* Yes, *dys* days, *yrs* Years

### Summary of clinical findings

In the cases reviewed in this study, P2-4 had relatively good VA despite severe fundus changes. The fundus changes in P1 were consistent with the grade 2 fundus lesions of NCMD, and the fundus changes in P2-4 were consistent with grade 3 (Fig. 3a, b, d, e) [7]. The fundus lesions of P2 did not change over a 6-year period, as reflected in fundus contrast (Fig. 3b, c). In addition, the inheritance pattern of this family was autosomal dominant (Fig. 1a) [2], and genetic analysis showed that there was whole gene duplication of the PRDM13 gene in P1-4 (Fig. 1b-d) [6]. Therefore, we believe that the diagnosis of MCDR1 in this Chinese family is correct. We summarized parts of the clinical manifestations of the patients in this study in Table 1 for ease of understanding.

## Discussion

PRDM13 is a member of a large family of “helix-loop-helix” DNV-binding proteins that play an important role in cell differentiation by regulating gene expression during development [13]. Using human iPSC-derived retinal tissue, Kent et al. demonstrated that the PRDM13 gene is the only gene involved in the regulation of retinal development at the MCDR1 locus [6]. In this study, the PRDM13 gene was expressed as a whole gene duplication, and the duplication of exons 1–3 was confirmed. Moreover, Manes et al. caused severe loss of the imaginal eye-antennal disc in Drosophila by overexpressing PRDM13 alone, demonstrating the importance of PRDM13 in retinal development in an animal model [14]. Therefore, abnormalities in PRDM13 play an important role in the pathogenesis of MCDR1, but the specific molecular mechanism remains to be elucidated.

Genetic counseling plays an important role in the diagnosis of NCMD patients. Since NCMD shares phenotypic characteristics with other macular degenerations, such as age-related macular degeneration (AMD), Stargardt’s disease, etc., the diagnosis of NCMD only based on clinical features may lead to misdiagnosis of NCMD [7, 9]. Genetic counseling can not only assist the diagnosis of the proband, but also identify the type of NCMD and the underlying genetic cause of the disease through whole-genome sequencing, thus providing the possibility for further treatment [2, 9]. For example, Clustered regularly interspaced short palindromic repeats/crispr-associated protein-9 nuclease (CRISPR/Cas9) system as a frontier tool of gene-editing tools has been studied in the treatment of retinal degenerative diseases [15]. Research on techniques such as reducing the overexpression of PRDM13 by CRISPR/Cas9 or autologous transplantation combining CRISPR/Cas9 with human-induced pluripotent stem cells may provide effective treatments for NCMD [15, 16].

Similar to previous studies, patients P2-4 had relatively good VA that was inconsistent with their fundus presentation and allowed them to maintain long-term vision [17, 18]. A possible cause for this may lie with the perifoveal fixation formation of the nasal macula, which functions via the microperimetry system [17, 19, 20]. This may also explain the formation exotropia in P2. However, this conclusion still needs to be verified.

In addition, in terms of clinical manifestations, NCMD and AMD share many similarities, such as drusen, geographic atrophy (GA), and choroidal neovascularization (CNV) [7, 21]. The pathogenesis of early AMD is still unclear, but it is generally believed that RPE dysfunction plays an important role in the formation of drusen, and the first signs of AMD appear in the choriocapillaris, Bruch’s membrane and RPE [22, 23]. The corresponding macular area of grade 1 and 2 lesions in NCMD showed drusen-like changes, as well as GA in previous studies, which is similar to early AMD. Moreover, in this study, OCT of P1 showed abnormalities in the RPE layer, while the neurosensory retinal layer was relatively normal (Fig. 5a). For grade 3 lesions, some researchers expected a similarity to staphyloma [11], but the corresponding ultrasound image did not find any outpouching of the sclera [7]. Furthermore, coloboma refers to an absence of tissue that has never been present [7], so the most likely mechanism for the formation of grade 3 lesions is atrophy. In addition, we also found that RPE and choroid atrophy in the macular caldera lesions was greater than that in the neurosensory retina, although some areas in the lesions were still covered by these layers. This feature has also been shown in OCT in previous studies but has not been described in detail (Fig. 6a-c) [7, 12]. The RPE and choroid are closely related during embryogenesis, and transgenic mouse experiments have shown that RPE deficiency can cause loss of choroid development [24]. Therefore, RPE and choroidal atrophy appear to play an important role in the macular dysplasia of NCMD. In macular caldera lesions, neurosensory retinal atrophy may be secondary to RPE and choroidal atrophy. The relatively retained neurosensory retina may also be part of the reason why NCMD patients maintain relatively good VA. Moreover, Kent et al. found that optical coherence tomographic angiography (OCTA) revealed that the retinal vascular structures remained relatively intact compared with FFA, which further supports this view [25]. In addition, RPE and choroidal atrophy may also be one of the causes of scleral dilation in some patients with macular caldera lesions (Fig. 5b). Choroidal atrophy can lead to ischaemia and hypoxia of the sclera, which leads to remodelling of the extracellular matrix of the sclera and ultimately to ectasia of the sclera [26, 27]. However, this view still needs to be further verified.

## Conclusions

In conclusion, RPE and choroidal atrophy appear to play an important role in the development of macular caldera. NCMD and AMD have many similar clinical manifestations. Although MCDR1 is rare, its study may help us to further understand the pathogenesis of AMD and the development process of the macula.

## Data Availability

The datasets used and/or analyzed during the current study are available from the corresponding author on reasonable request. The variant information of PRDM13 gene (NC_000006.11:g.100054862-100062667dup) of patients, in our study, has been submitted to the “Global Variome shared LOVD” (https://databases.lovd.nl/shared/individuals/00419091).

## Abbreviations

NCMD: North Carolina macular dystrophy
OCT: Optical coherence tomography
FAF: Fundus autofluorescence
mfERGs: Multifocal electroretinograms
ERG: Full field electroretinogram
VEP: Visual evoked potential
VA: Visual acuity
IOP: Intraocular pressure
RPE: Retinal pigment epithelium
FFA: Fundus fluorescence angiography
AMD: Age-related macular degeneration
CRISPR/Cas9: Clustered regularly interspaced short palindromic repeats/crispr-associated protein-9 nuclease
GA: Geographic atrophy
CNV: Choroidal neovascularization
OCTA: Optical coherence tomography angiography
